# National Surveillance of Legionnaires’ Disease, China, 2014–2016

**DOI:** 10.3201/eid2506.171431

**Published:** 2019-06

**Authors:** Tian Qin, Hongyu Ren, Dongke Chen, Haijian Zhou, Luxi Jiang, Duorong Wu, Jilu Shen, Fengyan Pei

**Affiliations:** Chinese Center for Disease Control and Prevention, Beijing, China (T. Qin, H. Ren, H. Zhou, L. Jiang);; State Key Laboratory for Infectious Disease Prevention and Control, Beijing (T. Qin, H. Zhou);; Collaborative Innovation Center for Diagnosis and Treatment of Infectious Diseases, Hangzhou, China (T. Qin, H. Zhou);; Beijing Hospital, Beijing (D. Chen);; Haikou People's Hospital, Haikou, China (D. Wu);; The Fourth Affiliate Hospital of Anhui Medical University, Hefei, China (J. Shen);; Jinan Central Hospital, Jinan, China (F. Pei)

**Keywords:** Legionnaires’ disease, bacteria, China, Legionella pneumophila, antimicrobial susceptibility, molecular subtyping, serogroup

## Abstract

We report national surveillance of Legionnaires’ disease in China. Urine samples from 11 (3.85%) of 286 patients with severe pneumonia of unknown cause were positive for the *Legionella pneumophila* serogroup 1 antigen. We isolated *Legionella* strains from 7 patients. Improved diagnostic testing is needed for this underestimated disease in China.

Legionnaires’ disease is a form of atypical pneumonia caused by bacteria of the genus *Legionella*. *L. pneumophila* serogroup 1 causes most Legionnaires’ disease (*1*). Although Legionnaires’ disease has been reported worldwide, only a few sporadic cases have been reported in China (*2*). Investigation of *Legionella* infection is urgently needed in China to describe its prevalence and epidemiology.

During 2014–2016, we conducted surveillance of Legionnaires’ disease in 18 hospitals in China under the coordination of the Chinese Center for Disease Control and Prevention (China CDC). The Ethical Committee of the National Institute for Communicable Disease Control and Prevention, China CDC (ICDC-2014009), provided ethics approval for this study. The distribution of the 18 hospitals accounted for all regions of China (Appendix Figure 1). The hospital's clinical diagnostic level, pneumonia pathogen detection level, and degree of cooperation with this investigation were also considered. All 18 hospitals are level 3 first-class general hospitals, representing the highest level of healthcare in their cities.

The 3,132 severe pneumonia cases were defined and detected according to the Guidelines for the Diagnosis and Treatment of Community-Acquired Pneumonia in Adults in China (2016 edition) (*3*) (Appendix Figure 2). Among them, 1,885 cases were diagnosed as noninfectious or nonbacterial infections, and 771 cases were diagnosed as bacterial infections other than *Legionella* by daily testing, including bacterial culture, viral nucleic acid detection, and immunologic detection in hospital laboratories. Patients with the remaining 476 cases of pneumonia with unknown cause were enrolled and tested for *Legionella* infection. Among them, 190 left the hospital, died, or were unwilling to cooperate. Thus, urine samples were collected from 286 patients and sent for urine antigen detection for *L. pneumophila* serogroup 1 (BinaxNow, https://www.alere.com) (Appendix Figure 2). Sputum samples were obtained from 211 of the 286 patients and sent to the laboratory of China CDC (Beijing, China) for *Legionella* culture, which used both buffered charcoal yeast extract agar and buffered charcoal yeast extract agar supplemented with *Legionella* GVPC (glycine, vancomycin, polymyxin, cycloheximide) Selective Supplement (Oxoid, https://www.thermofisher.com). Eleven (3.85%) of the 286 urine samples yielded positive results, and we isolated *Legionella* strains from 7 of them. All 7 *L. pneumophila* cultures were obtained from the same patients who tested positive by urine antigen detection. The positive rate of *Legionella* culture was 3.32% (7/211). All isolated *Legionella* strains were *L. pneumophila* serogroup 1.

All 11 urine antigen–positive patients were male, 23–76 years of age (average 56 years) (Table). They resided in 7 cities, and most (9/11) cases were observed in summer (in China, July–September). All were hospitalized; length of hospitalization ranged from 7 to 93 days. Six were admitted to an intensive care unit. The case-fatality rate was 18.2% (2/11) after antimicrobial and supportive therapies.

**Table:** Characteristics of 11 Legionnaires’ disease patients, China*

Patient ID	Age, y	Date of diagnosis	Onset city	Underlying illness	Length of hospitalization, d	ICU admission	Outcome	SBT type of isolates
1	45	2014 Sep	Shenyang	None	15	Yes	Recovered	ST2344
2	70	2014 Aug	Beijing	None	93	Yes	Recovered	ST59
3	53	2015 Aug	Hefei	None	18	No	Recovered	ST2369
4	63	2016 Jan	Jinan	None	16	No	Recovered	ST42
5	67	2016 Aug	Haikou	Diabetes	21	No	Recovered	ST742
6	23	2016 Jul	Beijing	AIDS	7	Yes	Died	ST2366
7	53	2016 Sep	Shanghai	None	14	Yes	Recovered	ST2368
8	58	2016 May	Lishui	None	8	No	Recovered	NS
9	49	2014 Jul	Shenyang	Cirrhosis	14	Yes	Died	NS
10	76	2015 Jul	Beijing	None	17	Yes	Recovered	NS
11	59	2016 Sep	Jinan	None	22	No	Recovered	NS

*All patients were male. ICU, intensive care unit; ID, identification; NS, no strain isolated; SBT, sequence-based typing; ST, sequence type.

We performed antimicrobial susceptibility testing using E-test strips (bioMérieux, https://www.biomerieux.com). According to the epidemiologic cutoff values of the European Committee on Antimicrobial Susceptibility Testing (*4*) or as determined by a previous study (*5*), all 7 strains were susceptible to fluoroquinolones, macrolides, and rifampin but not to cefuroxime (Appendix Table 1).

We subtyped the 7 strains using pulsed-field gel electrophoresis (*6*) and sequence-based typing (SBT) (*7*). All 7 strains were identified as different pulsed-field gel electrophoresis and SBT types (Appendix Figure 3). Submission to the European Working Group on *Legionella* Infections *L. pneumophila* SBT database (http://www.ewgli.org) identified 4 profiles as new; these profiles were assigned new sequence types (STs) (ST2344, ST2366, ST2368, and ST2369). Querying the European Working Group on *Legionella* Infections database showed that 2 STs (ST42 and ST59) contained strains that are distributed worldwide (Appendix Table 2). We also tested the 7 strains for their intracellular growth ability using previously described methods (*8*), and all showed high intracellular growth in J774 cells, suggesting that these strains are pathogenic (Appendix Figure 4).

Many Legionnaires’ disease cases are reported worldwide, including hundreds in the United States and Europe each year (*9*,*10*). However, no data are available on the prevalence of Legionnaires’ disease in China. In China, no *Legionella* urine antigen test reagent has been approved for clinical diagnosis and few hospitals conduct *Legionella* culture, so in clinical laboratories, Legionnaires’ disease is difficult to detect; therefore, diagnosis is based mainly on signs and symptoms. Legionnaires’ disease is usually diagnosed as unexplained pneumonia.

The results of this study showed that *L. pneumophila* is an important pathogen for pneumonia patients in China, and current diagnostic methods in China may misdiagnose or overlook it. We suggest establishment of a routine monitoring reporting system to investigate the prevalence and epidemiology of Legionnaires’ disease in China.

Appendix Additional information from a study of Legionnaires’ disease surveillance, China, 2014–2016.
