# Supplementary material for: National Surveillance of Legionnaires’ Disease, China, 2014–2016
Source: Emerg Infect Dis. 2019 Jun;25(6):1218–9. doi: 10.3201/eid2506.171431 (PMC6537748; doi:10.3201/eid2506.171431)
Supplement: Appendix — Additional information from a study of Legionnaires’ disease surveillance, China, 2014–2016. [file 17-1431-Techapp-s1.pdf]

Article DOI: https://doi.org/10.3201/eid2506.171431

# National Surveillance of Legionnaires' Disease, China, 2014–2016

## Appendix

**Appendix Table 1.** MICs results for 7 *Legionella pneumophila* strains, China, 2014–2016*

| | Antimicrobial drug, susceptible MIC, mg/dL | | | | | | | |
|---|---|---|---|---|---|---|---|---|
| Strain ID | CIP, <2.0 | LVX, <1.0 | MXE, <1.0 | ERY, <0.5) | AZM, <1.0 | CLR, <0.5 | RIF, <0.032 | CXM, <1.0 |
| CNLP001 | 0.125 | 0.094 | 0.38 | 0.094 | 0.094 | 0.125 | 0.016 | 3 |
| CNLP002 | 0.125 | 0.094 | 0.25 | 0.094 | 0.032 | 0.25 | 0.012 | 4 |
| CNLP003 | 0.5 | 0.094 | 0.38 | 0.094 | 0.047 | 0.125 | 0.032 | 1.5 |
| CNLP004 | 0.125 | 0.064 | 0.38 | 0.064 | 0.047 | 0.25 | 0.032 | 8 |
| CNLP005 | 0.25 | 0.094 | 0.38 | 0.064 | 0.032 | 0.125 | 0.023 | 2 |
| CNLP006 | 0.25 | 0.125 | 1 | 0.064 | 0.094 | 0.25 | 0.002 | 2 |
| CNLP007 | 0.25 | 0.125 | 0.38 | 0.125 | 0.094 | 0.125 | 0.023 | 3 |

* The MIC values with bold font are greater than the cutoff values of the specific antimicrobial agent. The MICs with normal fonts are < the cutoff values of the specific antimicrobial agent. AZM, azithromycin; CIP, ciprofloxacin; CLR, clarithromycin; CXM, cefuroxime; ERY, erythromycin; LVX, levofloxacin; MCE, moxifloxacin; RIF, rifampin.

**Appendix Table 2.** Number, sources, regional distribution, and year distribution of strains of STs determined in this study in the *Legionella pneumophila* SBT database

| | No. strains in SBT database | | | | |
|---|---|---|---|---|---|
| ST | Total | Clinical | Environmental | Regional distribution | Year distribution |
| ST-42* | 324 | 269 | 52 | 30 countries | 1982–2017 |
| ST-59 | 134 | 73 | 61 | 17 countries | 1982–2016 |
| ST-742 | 3 | 2 | 1 | 2 countries | 2009, 2013 |
| ST-2344 | 1 | 1 | 0 | 1 country | 2014 |
| ST-2366 | 1 | 1 | 0 | 1 country | 2016 |
| ST-2368 | 1 | 1 | 0 | 1 country | 2016 |
| ST-2369 | 1 | 1 | 0 | 1 country | 2015 |

*In the database, the sources of three ST-42 strains were unknown. SBT, sequence-based typing; ST, sequence type.

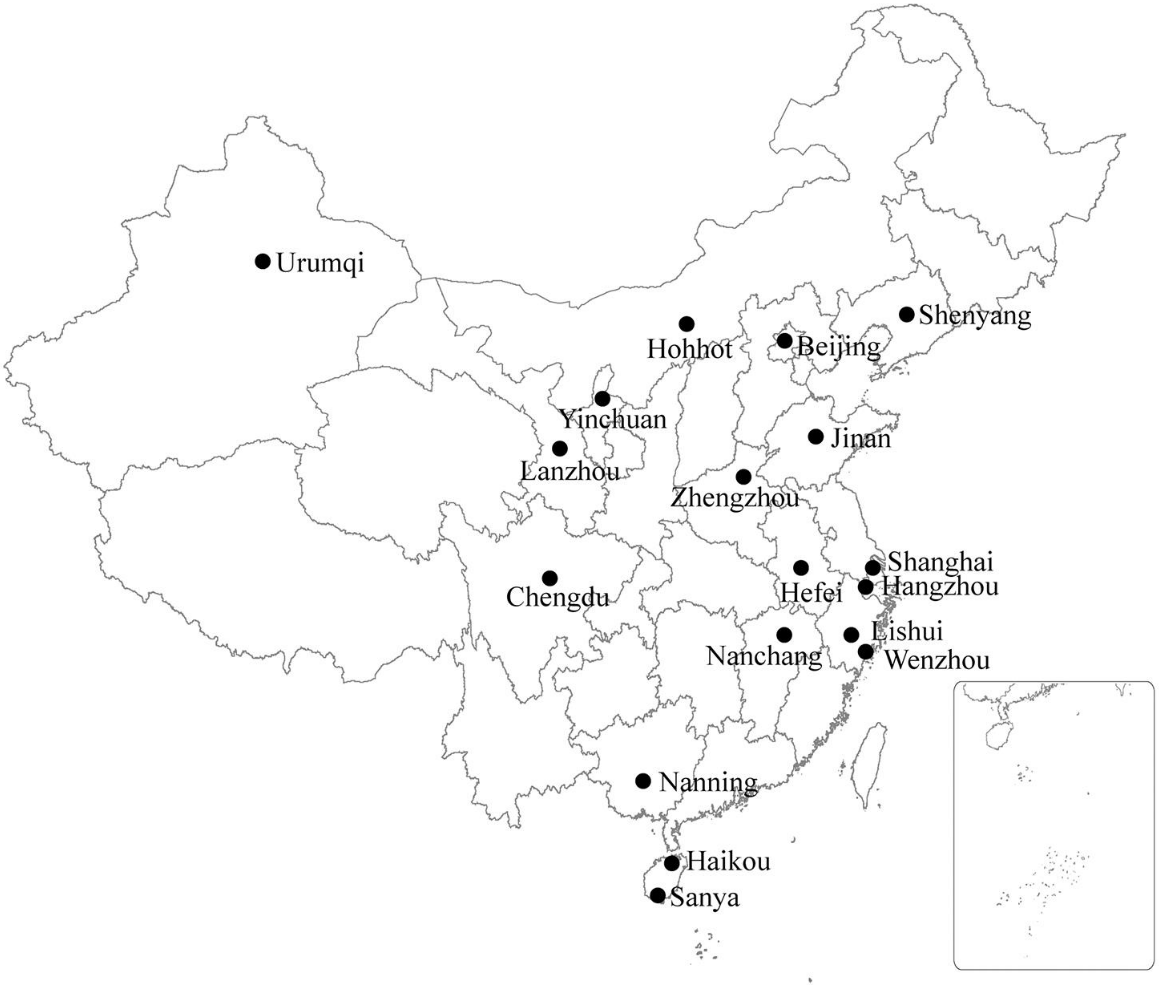


**Appendix Figure 1.** Geographic distribution of 18 hospitals (1 hospital was chosen in each city) in the national survey of *Legionella pneumophila* infections, China, 2014–2016.

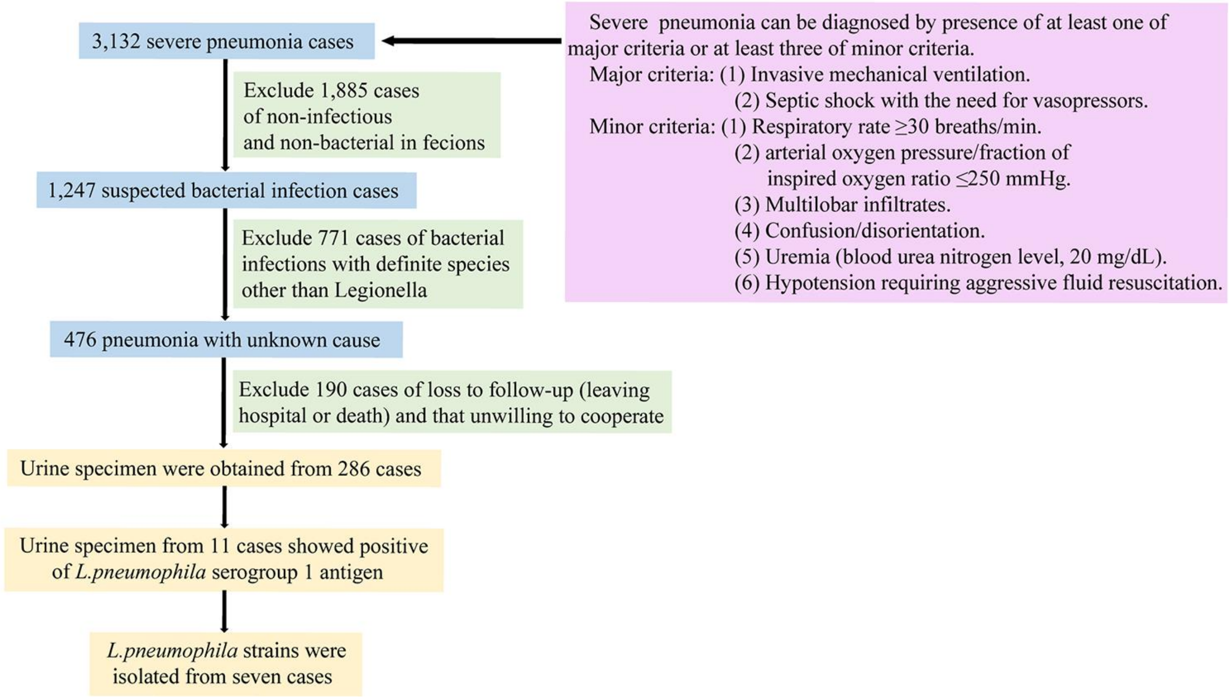


**Appendix Figure 2.** Screening procedure for Legionnaires' disease in the national surveillance in 18 hospitalsChina, 2014–2016.

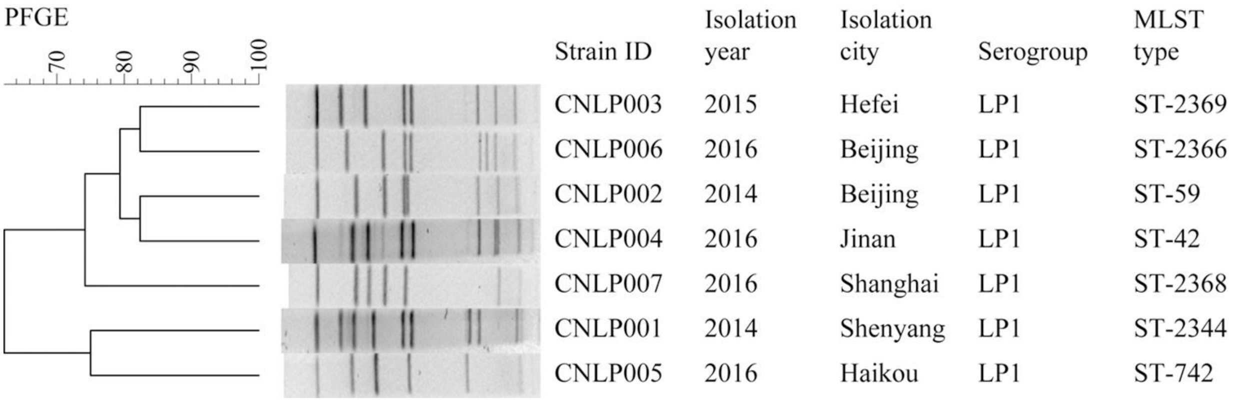


**Appendix Figure 3.** Clustering of the 7 clinical *Legionella pneumophila* strains based on pulsed-field gel electrophoresis patterns. The strain ID, isolation year, isolation city, serogroup, and multilocus sequence typing type of each isolate are listed to the right of the patterns.

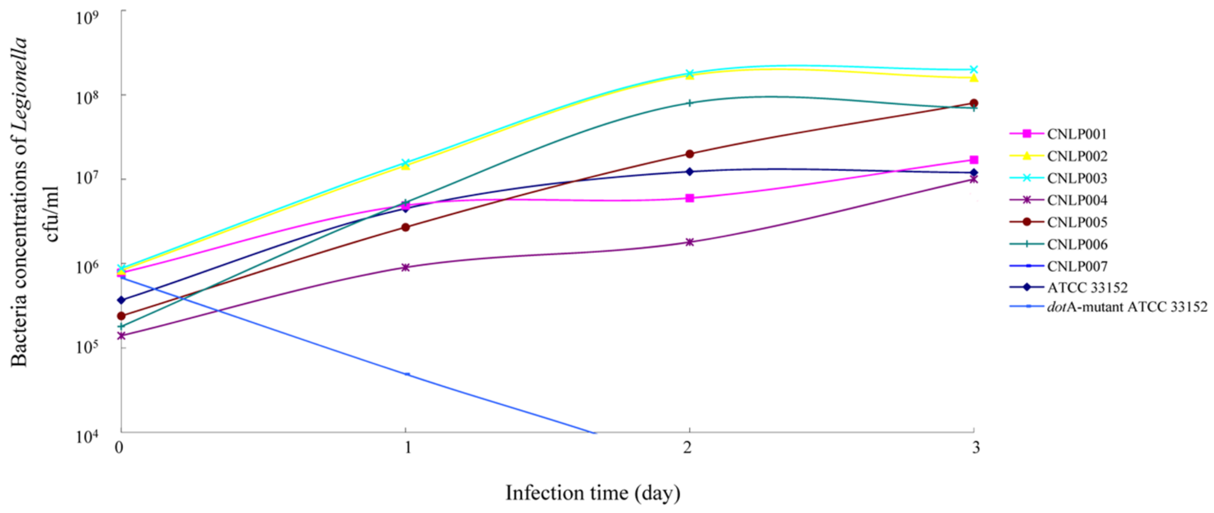


**Appendix Figure 4.** Intracellular growth ability of 7 clinical *Legionella pneumophila* strainsChina, 2014–2016. The formation of colonies (colony-forming units/mL) was determined in triplicate at the times indicated, and the representative data of at least 4 independent experiments are shown. The *L. pneumophila* philadelphila-1 strain ATCC33152 and its *dot*A mutant were used as positive and negative controls, respectively.
